# Complete Genome Sequence of Gordonia rubripertincta SD5, a Soil Bacterium Isolated from a Di-(2-Ethylhexyl) Phthalate-Degrading Enrichment Culture

**DOI:** 10.1128/MRA.01087-20

**Published:** 2020-11-05

**Authors:** Sang-Soo Han, Hye Kyeong Kang, Bok Yeon Jo, Byung-Gon Ryu, Hyun Mi Jin, Eu Jin Chung, Ji Young Jung

**Affiliations:** a Microbial Research Department, Nakdonggang National Institute of Biological Resources (NNIBR), Sangju-si, Gyeongsangbuk-do, South Korea; Queens College

## Abstract

We report the complete genome sequence of Gordonia rubripertincta SD5, isolated from a soil-derived di-(2-ethylhexyl) phthalate-degrading enrichment culture. The final genome assembly consists of a 5.10-Mbp chromosome and a plasmid (159 kbp). A total of 4,814 coding sequences were predicted, including 4,741 protein-coding sequences.

## ANNOUNCEMENT

Di-(2-ethylhexyl) phthalate (DEHP) is a phthalate ester (PAE); it is mostly used as a plasticizer for polyvinyl chloride (PVC) products. Because PAEs do not chemically bond with PVCs, PAEs are easily released into the environment, where they accumulate (1, 2). Given the detrimental effects on health and the environment, PAE contamination has caused public concern (3, 4). Members of the genus *Gordonia*, which belongs to the family *Gordoniaceae* in the phylum *Actinobacteria*, have been isolated from environmental sources such as soil, contaminated environments, industrial wastewaters, and human body habitats (5); some members can reportedly degrade PAEs, including DEHP (6–8). We isolated Gordonia rubripertincta SD5 from a DEHP-degrading enrichment culture obtained from soil and sequenced the genome to obtain fundamental insights into the metabolic pathways for DEHP degradation.

A soil sample was collected from the bank of the Gapcheon stream (36°26′16.4″N, 127°23′39.2″E) in Daejeon City, South Korea, and a soil-derived enrichment culture was obtained by incubating the sample at 30°C aerobically with shaking (180 rpm) using mineral salts medium with 500 ppm DEHP as the sole carbon source. The 4-week-cultured sample was diluted in phosphate-buffered saline (pH 7.4), and the dilutions were spread over Reasoner’s 2A (R2A) agar (Difco). After incubation at 30°C for 3 days, we obtained a single colony, named SD5. Using Maxwell 16 DNA purification kits (Promega), the genomic DNA was extracted from strain SD5 and grown in R2A broth (Difco) at 30°C. The DNA was sequenced using the PacBio RS II platform with a 20-kb SMRTbell library and the Illumina HiSeq X Ten platform (151-bp paired-end reads) with a 350-bp insert size at Macrogen, Inc. (South Korea). Sequencing libraries were prepared using a SMRTbell template prep kit v1.0 (PacBio) and a TruSeq Nano DNA library prep kit (Illumina). A total of 117,799 subreads (1.148 Gbp; coverage, 218.09-fold; mean subread length, 9,744 bp; *N*_50_, 14,167 bp) generated by PacBio RS II were used for *de novo* genome assembly using FALCON-integrate v2.1.4 (9). A total of 4,107,278 quality-filtered paired-end Illumina reads (0.62 Gbp; coverage, 117.84-fold), in which ≥90% of bases had a Phred score of 30 or above, were used for error correction using Pilon v1.21 to construct the final genome assembly (10). Then, the genome was annotated using NCBI PGAP v4.12 (11). Default parameters were used for all software unless otherwise specified.

The genome consisted of a circular chromosome (5,104,173 bp; GC content, 67.59%) and a circular plasmid designated pGRS1 (159,004 bp; GC content, 64.95%). The genome, assembly, and annotation statistics are shown in Table 1. Average nucleotide identity (ANI) analysis was conducted with OrthoANIu (12) for accurate identification of strain SD5 and resulted in 98.40% similarity with Gordonia rubripertincta NBRC 101908^T^ (GenBank accession numbers BAHB01000001 to BAHB01000134). The value over the ANI threshold range (95 to 96%) for species delineation (13) indicates that strain SD5 belongs to the same species. The NCBI PGAP predicted a total of 4,877 genes, including 4,741 protein-coding genes, 12 rRNA genes, 48 tRNA genes, 3 noncoding RNAs, and 73 pseudogenes. Functional annotation and KEGG pathway mapping were performed using BlastKOALA v2.2 (14). Of the predicted genes, 2,206 genes were categorized in 240 functional pathways; 25 and 107 genes that may be involved, respectively, in aromatic compound degradation and xenobiotic degradation and metabolism were detected. The genome information of Gordonia rubripertincta SD5 will greatly contribute to establishing the phthalate degradation pathway.

**TABLE 1:** Summary of the assembly and annotation statistics of Gordonia rubripertincta SD5

Genetic element	Genome size (bp)	GC content (%)	No. of coding sequences	No. of rRNAs	No. of tRNAs	Coverage (×)	GenBank accession no.
Chromosome	5,104,173	67.59	4,589	12	48	179	CP059694
pGRS1	159,004	64.95	152	0	0	142	CP059695
Total	5,263,177	67.51	4,741	12	48	177	

### Data availability.

The whole-genome sequence and raw sequencing reads for strain SD5 were deposited under GenBank accession numbers CP059694 and CP059695, BioProject accession number PRJNA649373, BioSample accession number SAMN15665176, and SRA accession numbers SRX8842321 and SRX8842322.
